# Local Piezoelectric Response of Polymer/Ceramic Nanocomposite Fibers

**DOI:** 10.3390/polym14245379

**Published:** 2022-12-08

**Authors:** Aurora Magnani, Simone Capaccioli, Bahareh Azimi, Serena Danti, Massimiliano Labardi

**Affiliations:** 1 Dipartimento di Fisica “Enrico Fermi”, Università di Pisa, Largo Pontecorvo 3, 56127 Pisa, Italy; 2 CNR-IPCF, Pisa Unit, Largo Pontecorvo 3, 56127 Pisa, Italy; 3 CISUP, Centro per l’Integrazione della Strumentazione dell’Università di Pisa, 56126 Pisa, Italy; 4 Dipartimento di Ingegneria Civile ed Industriale (DICI), Università di Pisa, L. Lazzarino 1, 56122 Pisa, Italy; 5 Department of Translational Research and New Technologies in Medicine and Surgery, University of Pisa, 56126 Pisa, Italy

**Keywords:** electrospun composite nanofiber, piezoelectric coefficient, piezoresponse force microscopy

## Abstract

Effective converse piezoelectric coefficient (*d*_33,eff_) mapping of poly(vinylidene fluoride) (PVDF) nanofibers with ceramic BaTiO_3_ nanoparticle inclusions obtained by electrospinning was carried out by piezoresponse force microscopy (PFM) in a peculiar dynamic mode, namely constant-excitation frequency-modulation (CE-FM), particularly suitable for the analysis of compliant materials. Mapping of single nanocomposite fibers was carried out to demonstrate the ability of CE-FM-PFM to investigate the nanostructure of semicrystalline polymers well above their glass transition temperature, such as PVDF, by revealing the distribution of piezoelectric activity of the nanofiber, as well as of the embedded nanoparticles employed. A decreased piezoelectric activity at the nanoparticle site compared to the polymeric fiber was found. This evidence can be rationalized in terms of a tradeoff between the dielectric constants and piezoelectric coefficients of the component materials, as well as on the mutual orientation of polar axes.

## 1. Introduction

Piezoelectrics represent a peculiar class of materials, in which mechanical and electrical effects are combined. By virtue of a mechanical deformation, electrical dipole moments can be created within the atomic cell (i.e., direct piezoelectric effect), and in contrast, application of electric fields can produce mechanical stress (i.e., converse piezoelectric effect). Piezoelectric devices have great technological importance, with application, e.g., as sensors, actuators, and energy scavengers [1,2], as well as in biomedicine [3]. The development of piezoelectric nanostructures has allowed their exploitation as nanocomposite materials, e.g., combining mechanical properties and electromechanical activity [4], or to increase the available specific surface, enhancing interfacial effects at the contact area to obtain electroactive biological scaffolds [3,5].

An interesting class of piezoelectric nanostructures is poly(vinylidene fluoride) (PVDF) piezoelectric nanofibers developed for biomedical applications [6,7]. Due to their properties, including thermal stability and chemical inertness, PVDF and its copolymers have shown good compatibility with human body tissues, and have increasingly attracted attention in new-concept biomedical devices [7].

Electrospinning is a well-known, simple and highly tunable method for ultrafine and nanofiber fabrication from polymer solutions, which is conveniently used for producing PVDF with a high content of the ferroelectric β-phase crystals by inherently providing both mechanical stretching and electrical poling [8]. In addition, nanocomposite piezoelectric fibers can be produced by their incorporation in polymeric solutions of ceramic nanoparticles, which may result in the enhancement of the overall piezoelectric properties, and by the choice of suitable piezoelectric ceramic nanoparticles, such as BaTiO_3_, LiNbO_3_ and ZnO [6,9,10].

Characterization of the piezoelectric functionality of piezoactive nanostructures on the local scale can be crucial to rationalize the overall behavior of the produced scaffolds in terms of the piezoelectric performance of the polymer, its modification due to the embedded nanoparticles, as well as of its surface. To this purpose, methods based on atomic force microscopy (AFM) can be applied to detect local surface deformations due to the converse piezoelectric effect. This is obtained by the application of an electric potential to the AFM conductive probe tip that has a nanometer-sized edge and is generally attached to a flexible cantilever, which is devised to detect forces applied to the tip by means of its deflection, and is therefore sensitive to surface deformations.

In the AFM contact mode, the probe is in continuous contact with the surface. When the material is piezoelectric, the applied electric field inside the sample induces a surface deformation that displaces the tip and translates into deflection of the cantilever. Such a method is generically termed as contact-mode piezoresponse force microscopy (PFM). Enhanced sensitivity to small surface deformations can be obtained by exploiting the existence of a cantilever resonance in contact conditions, which is able to boost the response to tiny displacements [11]. Refined state-of-the-art variants are available to separate themselves from changes in such resonance due to the local mechanical properties of the surface, e.g., the dual AC resonance tracking (DART) method [12]. Yet, these methods still rely on a continuous contact between the tip and surface; therefore, their ideal performance is obtained with stiff materials, as well as with nanostructures strongly bound to a substrate. On the other hand, sideways dragging of nanoparticles or nanofibers laid down on the substrate is often difficult to avoid in contact mode, preventing the proper examination of this kind of sample.

Literature studies of piezoelectric polymers investigated by local probe methods such as PFM are not numerous. In particular, concerning electrospun PVDF fibers such as those studied in this work, PFM maps are reported only in a few cases. Baji et al. [13] analyzed the piezoelectric response of a BaTiO_3_ nanofiber made up of self-assembled grains, and cladded by PVDF, and showed a variable local response with phase reversal spots of the piezoresponse along the fiber, probably indicating domains or nanoparticles with opposite polar orientation. Sencadas et al. [14] visualized a higher PFM signal at the sides of the fibers than at its top surface, which is plausible due to the tip shape effects, along with a homogeneous response on the top of the fiber, with no particular evidence of piezoelectric domains. The difficulty in visualizing polydomain structures in PVDF could be due to its low glass transition temperature (*T*_g_) of around −40 °C, so that the amorphous phase likely surrounding the crystallites, being much softer than the crystals themselves, may hinder or average out the detection of the piezoelectric strain of the single crystallites.

Liu et al. [15,16] evidenced some domain structure on top of the electrospun fibers, and reported an increase in the effective piezoelectric coefficient (*d*_33,eff_) with a decrease in the fiber diameter. Ico et al. [17] found a rather uniform PFM signal, with a longitudinal region at the nanofiber top, with a different PFM amplitude and phase contrast. An increase in the piezoresponse by decreasing nanofiber size was also evidenced.

The piezoelectric polydomain structure of PVDF-trifluoroethylene (TrFE) could be clearly evidenced by PFM on ultrathin crystalline films grown on doped silicon substrates [18], since in this case no amorphous phase was present, and the mechanical properties of such crystals were favorable for the optimal performance of contact-mode PFM. Polydomain structures were also reported on polyhydroxybutyrate (PHB) nanofibers [19], where the *T*_g_ of PHB (5–20 °C) most likely corresponds to a stiffer amorphous phase, favoring the performance of contact-mode PFM.

Piezoresponse characterizations on PVDF electrospun nanofibers have been often conducted based on local polarization loops, obtained by placing the probe at a fixed position on the fiber top, and ramping the DC-applied potential while recording the PFM response [13,16,20,21,22,23,24]. However, it is still matter of debate whether the butterfly loops typically recorded on ferroelectrics are sufficient evidence to demonstrate piezoelectricity [25,26]. Other demonstrations rely on the linearity between the PFM signal and AC potential applied at fixed positions [27,28,29]. This method can also be disputed, since the electrostatic side-effects, as well as phenomena of different kind that could contribute to PFM signals (e.g., flexoelectricity and electrochemical strain [26]), have a linear trend with the applied potential.

A particular consideration should be given to the electrostatic side-effect of PFM mentioned above. In essence, when an AC electric potential of the form *V*(*t*) = *V*_dc_ + *V*_ac_ cos(Ω*t*), with Ω being the AC frequency, is applied between the probe and the sample, an electrostatic force *F*_el_(*t*) is induced on the probe, of the form *F*_el_(*t*) = *F*_dc_ + *F*_Ω_ cos(Ω*t*) + *F*_2Ω_ cos(2Ω*t*). Such force tends to bend the cantilever by Δ*z*_el_(*t*) = *F*_el_(*t*)/*k*, where *k* is the cantilever spring constant. A displacement of the surface due to the piezoelectric effect has the form Δ*h*_p_(*t*) = Δ*h*_p_ cos(Ω*t*), since the piezoelectric effect is linear with the applied potential, and produces a bending Δ*z*_p_(*t*) = Δ*h*_p_(*t*), since the probe in contact mode is constrained to the surface by the atomic forces in repulsive contact conditions. Since the component at frequency Ω of the cantilever bending is the one being detected in contact-mode PFM, in order to measure the piezoelectric displacement, the term *F*_Ω_ of the electrostatic contribution occurs at the same frequency of the piezo displacement, although the cantilever bending is caused in this case by electrostatic forces and not by the surface displacement. The ratio of the electrostatic side-effect to the genuine piezo displacement amounts to *F*_Ω_/*k*Δ*h*_p_. Therefore, there are two strategies to suppress the electrostatic side-effect: the first is to nullify *F*_Ω_ by the application of an appropriate value of *V*_dc_, and the second is to increase the value of the spring constant. The first strategy is tightly connected to so-called Kelvin probe force microscopy (KPFM) [26], where a position-dependent value of electric potential, named contact potential difference (*V*_CPD_), is measured by nulling the term *F*_Ω_ caused by the application of the opposite potential *V*_dc_ =−*V*_CPD_ to the probe during the scan. Since this method requires an independent measurement of *V*_CPD_ in order to operate the nullification of *F*_Ω_, its employment is not straightforward. The second strategy produces an overall suppression of the electrostatic side-effect, by employing cantilevers with much higher *k* than in contact mode, and is the one adopted in this work, described in the following.

Recently, an alternative PFM operation mode, named constant-excitation frequency-modulation (CE-FM)-PFM, has been introduced [30], showing the ability to obtain quantitative results for the local converse piezoelectric coefficient, not being limited to stiff materials and nanostructures, with a relatively simple measurement technique that is based on the intermittent-contact mode of the AFM. In CE-FM-PFM, the change in the cantilever oscillation amplitude *A* due to the surface displacement is measured, to obtain the amount of piezoresponse, instead of the cantilever bending. The advantages of this method are: (i) the average force exerted on the surface is far smaller than in the contact mode, enabling the investigation of softer materials than possible in contact mode, as well as facilitating the operation on weakly fastened nanoparticles; (ii) the cantilevers employed for dynamic modes are much stiffer (spring constant ~ 50 N/m) than the ones suitable for the contact mode (0.5–5 N/m), thereby operating the suppression of electrostatic side-effects as mentioned above. The drawbacks of CE-FM-PFM are instead: (i) the higher measurement noise, being a non-resonant method; (ii) the necessity to use rather low frequencies of the AC electric potential, of the order of 10–100 Hz, slowing down the measurement speed compared to contact-mode PFM; and (iii) the inability to perform PFM in the lateral direction, as possible in contact-mode PFM which profits from friction phenomena.

In this study, we apply CE-FM-PFM to investigate the local piezoelectric response of PVDF nanofibers containing BaTiO_3_ nanoparticles obtained via electrospinning. As already mentioned, this particular system was chosen as a typical example of nanostructures produced for applications both in the energy and biomedical fields. Two different design approaches towards the obtainment of improved piezoelectric performance in composites for biomedical or other applications can be envisaged. In both cases, the matrix should be biocompatible and/or have mechanical properties suitable for specific application. The nano-inclusions should deliver the enhancement of the piezoelectric performance, especially when the matrix itself is not electromechanically active, with a relatively low volume fraction, relying on the fact that nanoparticles exhibit a large surface to volume ratio, and therefore are expected to provide larger modifications of the properties of the matrix compared to a standard blend of the same materials. The case of PVDF (or its copolymers formulated to improve piezoelectric yield, e.g., PVDF-TrFE) as the matrix is largely validated in the literature [5,6,7], while the insertion of biocompatible piezoelectric nanoparticles is an assessed strategy to boost piezoelectric performance of these kinds of scaffolds. Nonetheless, non-piezoelectric matrix materials with mechanical properties optimized for specific applications could be used as well; in such cases, electromechanical activity will be provided only by nano-inclusions. In the Discussion section, a simple picture that is able to describe both situations from the point of view of the piezoelectric performance of composites will be sketched, and PFM appears a suitable method to validate the conclusions drawn from such a picture. Hence, the presented investigations should be regarded as a case study to demonstrate the capabilities of the alternative CE-FM-PFM local characterization technique, rather than being aimed at assessing the properties of nanostructured systems optimized for applications.

## 2. Materials and Methods

### 2.1. Nanofiber Fabrication

Composite PVDF/BaTiO_3_ nanofibers studied in this work were obtained by the following procedure. Commercially available PVDF (Solef® 1010, Solvay, Brussels, Belgium; M_n_ = 153 kDa, polydispersity index 2.3) was dissolved in dimethyl formamide (DMF)/acetone (2:1 *v/v*) at a polymer/solvent concentration of 20% *w/v* and stirred at 300 rpm for 12 h at room temperature. Commercially available BaTiO_3_ nanoparticles (100 nm diameter, from Sigma Aldrich) were dispersed in acetone at a nanoparticle/solvent concentration of 6% *w/v* by bath sonication for 45 min and successive probe sonication at 30 W for 30 min. Afterwards, the two solutions were mixed (3:1 *v/v*) and stirred for 45 min, to obtain a final concentration of 1:1 *v/v* DMF/acetone, 15% *w/v* PVDF/solvent, and 1.5% *w/v* BaTiO_3_/solvent. The polymeric solution with dispersed nanoparticles was loaded into a 10 mL glass syringe, fitted with a blunt tip stainless steel needle (21G × 3/4”) and placed into a syringe pump (NE-300, New Era Pump Systems, Inc., NY, USA). A cylindrical collector with 8 cm diameter (Linari Engineering s.r.l., Pisa, Italy), was placed at 15 cm distance from the tip of the needle. Positive potential of 35 kV from a HV power supply (S1600079; Linari High Voltage, Linari Engineering s.r.l., Pisa, Italy) was applied to the collector, while the needle was grounded. The polymer solution was injected from the needle in the presence of an electric field at a constant flow rate of 0.1 mL/h, directing the polymeric jet onto the collector, rotating at a speed of 2000 rpm. All the fabrication steps were performed at room temperature with a relative humidity of about 46%. The fiber meshes were kept in an oven at 60 °C for 12 h to remove residual organic solvent.

The present scaffolds were produced employing as-purchased BaTiO_3_ nanoparticles, in the framework of a more extended investigation using nanoparticles of different nature and with different surface functionalization, to be reported in future publications. The produced nanofibers were used here as a prototypical system to assess the ability of CE-FM-PFM to characterize local piezoelectric properties of this typology of nanostructures.

### 2.2. Nanofiber Characterization

The morphology of the produced fiber meshes was analyzed by field emission scanning electron microscopy (FE-SEM) using a FEI FEG-Quanta 450 instrument (Field Electron and Ion Company, Hillsboro, OR, USA). The samples were sputtered with platinum before analysis.

A systematic analysis of the morphology and physico-chemical properties of the obtained nanocomposites is out of the scope of this work. Indeed, only one typology of electrospun nanocomposites was studied here. In particular, standard structural characterization of the obtained nanofibers, as well as of the employed nanoparticles, by, e.g., XRD, IR, and Raman spectroscopy were not performed for our samples, since the analysis of the correlation between the production route, the obtained structure of the scaffolds, and their piezoelectric performance was not the aim of the present work, which is different from other works, e.g., Ref. [6]. In this work, instead, the assessment of the ability of CE-FM-PFM, as a scanning probe method alternative to customary contact-mode PFM, to determine the local piezoelectric performance of compliant samples, for instance amorphous polymers well above their glass transition temperature, is the main goal. Furthermore, standard TGA and DSC thermography were performed to corroborate the findings of local probe investigations. In particular, TGA with air flux provided an inorganic (BaTiO_3_) weight fraction of 3%, corresponding to a volume fraction of 1%, for the investigated fibers (Figure S1 of Supplementary Materials). DSC thermograms provided 68% crystalline fraction for the as-obtained polymer fibers, increasing to 72% after melting and recrystallization (Figure S2 of Supplementary Materials).

The used AFM was a NanoScope IIIa with a MultiMode head, equipped with a gas cell and ADC5 extension (Veeco Metrology, Santa Barbara, CA, USA), adapted to the CE-FM-PFM method as follows. Non-contact-type AFM cantilevers (Nanosensors PPP-NCLPt, platinum-iridium coated silicon tips, spring constant ~40 N/m, resonant frequency *ω*_0_ ~ 156 kHz, quality factor *Q*_0_ ~ 500 in air, tip radius ~30 nm) were operated in constant-excitation frequency modulation (FM)-AFM mode [31], with a free oscillation amplitude of *A*_0_ ~ 20 nm. Distance stabilization is obtained by feedback on the probe oscillation amplitude. An oscillating voltage *V*(*t*) = *V*_dc_ + *V*_ac_ cos(Ω*t*) is applied to the probe as is customary in PFM, with a typical AC electric potential frequency Ω of 85 Hz, *V*_dc_ = 0 V, and *V*_ac_ = 2 V_RMS_. The conductive sample substrate is connected to ground. The oscillation amplitude signal *A_ω_*(*t*) from the FM-AFM controller (PLLProII, RHK Technology, Troy, MI, USA) is demodulated at frequency Ω by a dual lock-in amplifier (SRS830DSP, Stanford Research Systems, Sunnyvale, CA, USA) whose amplitude output (Δ*A*_Ω_), as well as its phase ϕ_Ω_, was acquired through the auxiliary acquisition channels of our AFM. As detailed in Refs. [30,32], describing the implementation of the chosen method, the output Δ*A*_Ω_ represents the measurement of the surface displacement induced by the applied electric field, and therefore of the converse piezoelectric effect. By dividing such surface displacement by the applied voltage, an effective value of the piezoelectric coefficient *d*_33,eff_ is obtained. In the context of nanoscale measurement, effective *d*_33_ has the meaning of the local sample displacement detected by the atomic force probe in the direction normal to the surface, obtained when an electric voltage is applied between the probe and sample. It is known that numerous different electromechanical effects can contribute to the effective *d*_33_ value, in addition to the piezoelectric effect, depending on the sample typology, environmental conditions, and local scanning probe method used [26].

For PFM analysis, electrospun fibers were mechanically transferred by gentle contact onto a monocrystalline silicon substrate, which was connected to ground. Occasionally, single PVDF nanofibers were studied as formed when deposited directly on the electrospinning aluminum substrate. This situation was obtained by removing the nanofiber mesh from its substrate, whereby only the fibers adhering to aluminum, that is, the first ones to be deposited, remained exposed.

## 3. Results

**Figure 1:**
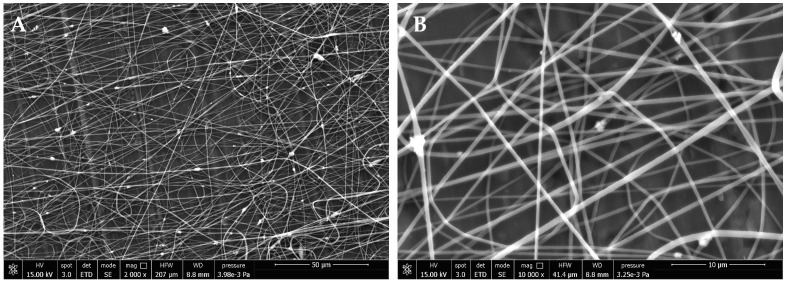
FE-SEM micrographs of the electrospun PVDF nanofiber mesh with dispersed BaTiO_3_ nanoparticles. Images were acquired at 15 kV and different magnifications: (**A**) 2000× (scale bar 50 µm), and (**B**) 10,000× (scale bar 10 µm).

Typical SEM micrographs of the obtained samples are shown in Figure 1. The predominant alignment direction (horizontal) of the fibers can be noticed in Figure 1A, due to the rotating collector technique, while vertical lines in the background are grooves of the aluminum foil used as the substrate. Overall, randomly oriented fibers are present in our sample. The obtained volume fraction of nanoparticles vs. polymer was estimated from image analysis of Figure 1A as 0.7%, which is consistent with expectations. Most of the inclusions appear to have the form of aggregates of a few nanoparticles (Figure 1B), although it was likely to have even single nanoparticles embedded in the fibers, as also evinced from AFM analysis.

**Figure 2:**
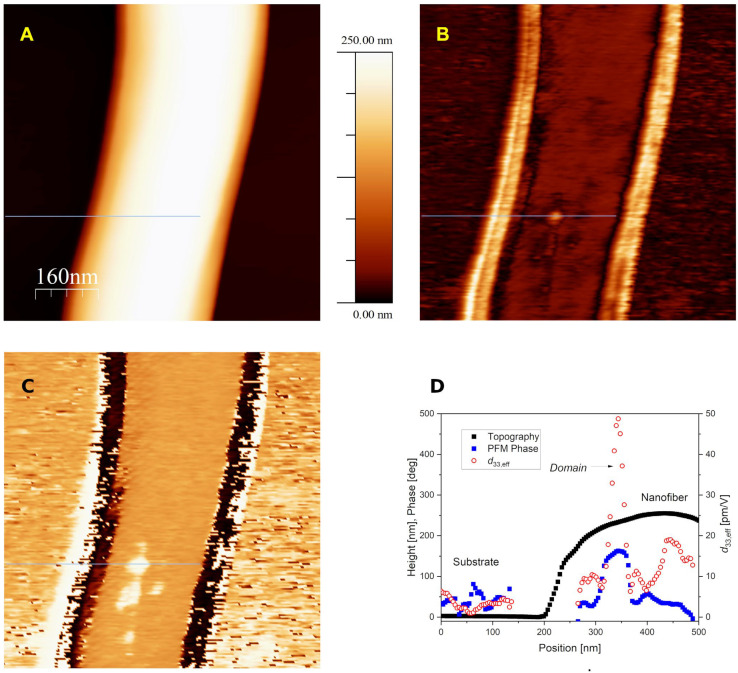
(**A**) Topography and CE-FM-PFM amplitude (**B**) and phase (**C**) scan of a portion of electrospun PVDF/BaTiO_3_ composite nanofiber, transferred on a doped silicon substrate. Bands on the flanks of the nanofiber in (**B**,**C**) should be disregarded, since they are caused by a spurious tip shape effect on the sloped region. Nanodomains show up on the fiber upper surface, where PFM phase is inverted. (**D**) Line profile corresponding to the horizontal stroke in (**A**–**C**), whose length is 500 nm. Data corresponding to the sloped region of the nanofiber have been excluded from the plot for clarity. Image size is 785 nm × 785 nm. The same color bar used in image (**A**) also defines the values of image (**B**) (0/100 pm/V) and (**C**) (−180°/+180°).

In Figure 2, AFM topography (A) and corresponding CE-FM-PFM amplitude (B) and phase scan (C) of a single nanofiber (250 nm diameter) is shown. From a representative line profile (Figure 2D), values of the amplitude modulation Δ*A*_Ω_ (PFM amplitude) measured on the fiber upper surface are consistent with expectations (10–20 pm/V) Much higher values were recorded on its sides (not shown for clarity in Figure 2D). This is a general feature of scanning probes applied to samples with steep topographic reliefs, since it is well known that the probe sides, rather than the tip edge, may contact the surface at steep slopes, giving rise to tip shape artifacts [33]. All measured quantities are prone to the same effect, so that their values should not be considered as reliable at the slopes of the structure. Therefore, only the results obtained at the nanofiber top will be considered here. An averaged effective piezoelectric coefficient *d*_33,eff_ ~ 13 pm/V was obtained, that is consistent with the literature data for the semicrystalline PVDF containing the ferroelectric β phase [13,17,27].

Ferroelectric nanodomains were also detected in a longitudinal region on the nanofiber top. Such domains show inverted PFM phase ϕ_Ω_ (Figure 2C), and a variety of piezoresponse values, even higher than the average of the nanofiber one. A line profile to evidence one of the domains with a higher piezoresponse, with *d*_33,eff_ ~ 50 pm/V, is shown in Figure 2D. The evidenced longitudinal nanodomain structure looks similar to the one reported in Ref. [17]. Average values of piezoresponse in the different regions of the sample are reported in the Supplementary Materials (Figure S3, Table S3).

The average piezoresponse value recorded on the substrate is around 6 pm/V, which could be partially due to residual electrostatic background, although the CE-FM-PFM method should be much more immune from electrostatic artifacts than contact-mode PFM [30]. There, background electrostatic PFM signal from the substrate is often observed when not intentionally compensated for by the application of a suitable DC bias, in addition to the AC potential needed for PFM detection. On the contrary, all data reported in this work where obtained by applying *V*_dc_ = 0 V, while still obtaining reasonably low electrostatic side effects. The employment of much stiffer force sensors, such as tuning fork quartz crystals (*k* ~ 10 kN/m), with the CE-FM-PFM technique, was demonstrated to completely suppress electrostatic side effects. This was also true with a PVDF thin film sample [32].

**Figure 3:**
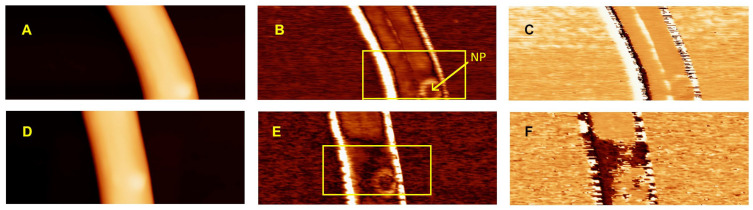
Topography (**A**), CE-FM-PFM amplitude (**B**) and phase (**C**) scan of an electrospun PVDF/BaTiO_3_ composite nanofiber deposited on doped silicon, where an embedded BaTiO_3_ nanoparticle is present (NP), indicated by the arrow. Nanodomains appear at the center of the fiber upper surface, where the PFM phase is inverted. The nanoparticle shows lower piezoresponse, while its border shows a higher signal. Topography (**D**), CE-FM-PFM amplitude (**E**) and phase (**F**) of the same nanofiber portion after poling by application of a DC potential to a region indicated by the rectangle in (**B**,**E**). Phase inversion is obtained after poling on the PVDF nanofiber, as well as on the nanoparticle and part of the nanodomains. The scan size is 1.28 μm × 0.5 μm. The same color bar of the image of Figure 2A defines the scale for the values of topography images (**A**,**D**) (0/250 nm), PFM amplitude (**B**,**E**) (0/50 pm/V) and phase (**C**,**F**) (−180°/+180°).

Figure 3 shows a different portion of nanofiber, where an embedded BaTiO_3_ nanoparticle was present, indicated by an arrow. Domains with an inverted phase are also present in this portion of the nanofiber, like the ones in Figure 2, with diverse amplitude values, both higher and lower than the average (Figure 3B,C). The center of the nanoparticle shows a value of *d*_33,eff_ ~ 8 pm/V (Figure 3B), smaller than the surrounding polymer, while a higher piezoresponse, of around 20 pm/V, was evidenced at its borders. The PFM phase at the nanoparticle had a similar value to that of the polymer nanofiber, while the nanoparticle borders have some spots with an inverted phase (Figure 3C). Incidentally, this higher piezoresponse could indicate peculiar properties at the interface between the polymer and the ceramics. The analysis of such interface effects is beyond the aims of the present work; however, these kinds of interface phenomena are crucial in the field of nanocomposites, and will be investigated more deeply in future works.

A poling experiment, similar to the one performed in Ref. [14], was conducted, by application of a DC potential of −15 V to the probe, while scanning the nanofiber area indicated in Figure 3B, including the BaTiO_3_ nanoparticle, and afterwards repeating the PFM imaging of the same region. Figure 3E shows the change in the PFM amplitude, while Figure 3F evidences the occurrence of a phase inversion. Therefore, it seems that the applied electric field was able to switch the polarization direction of both PVDF and the nanoparticle, since the PFM phase appears inverted on both. This result looks reasonable, since the applied potential corresponds in our case to an electric field of ~ 75 MV/m, higher than the coercive field of PVDF [34]. The scanning rate used for the poling stage corresponds to a residence time of the tip onto the nanofiber of ~ 6 ms/nm^2^. 

**Figure 4:**
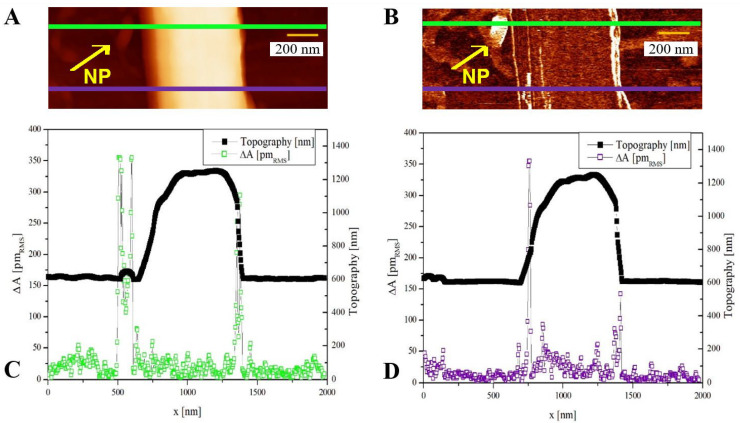
(**A**) Topography and (**B**) PFM amplitude scan of an electrospun PVDF/BaTiO_3_ composite nanofiber as deposited on the rotating electrospinning aluminum substrate, on which a BaTiO_3_ nanoparticle is also visible, indicated by the arrow. (**C**) Line profiles corresponding to the green line in (**A**,**B**), crossing the nanoparticle. (**D**) Line profiles corresponding to the purple line in (**A**,**B**), not crossing the nanoparticle. The applied potential *V*_ac_ was 1 V_RMS_ here; therefore, the Δ*A* value corresponds to the piezoelectric coefficient.

In a further example, Figure 4 shows the AFM topography and corresponding CE-FM-PFM scan of a single PVDF nanofiber (600 nm diameter) as formed when deposited directly on the electrospinning aluminum substrate.

A thin polymer layer (10–20 nm) was also found to be present on part of the substrate, probably a residual of removed fibers, showing the same piezoelectric response as the nanofiber itself. Additionally, one spot with a much higher piezoelectric performance, most likely an isolated BaTiO_3_ nanoparticle with around 50 nm diameter (indicated by arrows in Figure 4), was present on the substrate. The measured value of around 60 pm/V on this particle (see Figure S4 and Table S4 of Supplementary Materials) is consistent with PFM measurements on ~50 nm size nanoparticles from the literature [35]. This situation is not typical of the polymeric scaffolds, where nanoparticles are embedded within, or at least adhering to, the polymeric nanofiber; however, the case of a nanoparticle not embedded in the polymer, showing a higher piezoresponse than the embedded ones, could serve as a comparison within the interpretative framework sketched in the following Discussion section.

## 4. Discussion

Piezoelectric nanofibers are becoming a widely studied class of nanomaterials with different applications, ranging from energy harvesting, sensors, and biomedical devices able to impart local electrical stimuli to cells and tissues upon mechanical action [36,37]. Recent studies have underpinned the advancements in piezoelectric composite materials to achieve the best performing output in biomedicine [38]. Therefore, the local quantification of the piezoresponse has become impellent to understand the signals transmitted at a cell level. To address this question, we applied CE-FM-PFM to PVDF nanofibers incorporating barium titanate nanoparticles as a filler, which are a well-assessed configuration for the aforementioned applications.

In our measurements, piezoresponse at the location of nanoparticles is generally found to be lower than that of the surrounding polymer, whereas it was observed to be higher, or of similar magnitude, only occasionally. This behavior is consistent with the expected performance of the employed as-purchased BaTiO_3_ nanoparticles. This can be evinced from the declared value of their dielectric constant (150), whereas the tetragonal ferroelectric phase of a bulk BaTiO_3_ single-domain crystal should exhibit a dielectric constant of between 200 and 4600 at room temperature [39], depending on the crystal orientation. Measurements of the dielectric constant of these particles by broadband dielectric spectroscopy turned out in the range 38–76, decreasing with frequency [40], which is much smaller than the declared value, and even smaller than the measured values, at all temperatures, of both tetragonal and orthorhombic ferroelectric phases of bulk crystals [39]. Characterization of the piezoresponse of BaTiO_3_ nanoparticles is poorly documented in the literature [35,41], providing values of piezoelectric constants, determined by PFM, smaller than 70 pm/V, with increasing values when decreasing particle size.

In Figure 3, a lower value of piezoresponse is observed at the location of the embedded nanoparticle, and this result was generally obtained in several other scans (not shown). Such piezoelectric performance could be due to a low piezoelectric activity of the employed nanoparticles, and/or to a different spatial orientation of the polarization axis of the polymer, as well as of the nanoparticle, compared to the direction of the applied electric field.

A general picture to interpret these results should consider the effect of both the piezoelectric coefficient and the dielectric constant of the matrix (i.e., the nanofiber) and nano-inclusion (i.e., the nanoparticle). A simple model is sketched here to understand the role of dielectric constants and piezoelectric coefficients in composite materials. Let us start with the case of a BaTiO_3_ crystal of thickness *h* in a planar geometry, contacted by metallic electrodes with potential difference *V*. The electric field *E*_c_ inside the crystal amounts to *V*/*h*, and the related piezoelectric deformation Δ*z*_c_ = *d*_c_*V*, with *d*_c_ the piezoelectric coefficient of the crystal in the direction of the electric field. In the case of a polymer placed between the electrodes, the electric field *E*_p_ inside the polymer would amount to *V*/*h* as well, while its deformation would be Δ*z*_p_ = *d*_p_*V*, with *d*_p_ the piezoelectric coefficient of the polymer. In case both a crystal layer and a polymer layer were present, on top of each other (series configuration), with the first electrode on the side of the crystal, and the second one on the side of the polymer, the system should be regarded as a capacitor partially filled with two different dielectrics. If *h*_c_, *h*_p_ are the values of thickness of the two layers, for a total thickness *h*, the electric field in the crystal would drop, from elementary electrostatics, according to Equation (1), to:

(1) $$E_{c}=\frac{V}{\left(h_{c}+\frac{\varepsilon_{c}}{\varepsilon_{p}}h_{p}\right)}$$

where *ε*_c_ and *ε*_p_ are the relative dielectric constants of the crystal and polymer, respectively. If *ε*_c_ ~ 1000 (order of magnitude), as with BaTiO_3_ in the ferroelectric tetragonal crystal phase that exhibits the highest piezoelectricity and is stable at room temperature, and *ε*_p_ ~ 10, as with semicrystalline PVDF, the electric field in the crystal drops by a factor ~50 with respect to the case of the sole crystal, if *h*_c_ = *h*_p_ = *h*/2. Models for the effective dielectric constant of composite materials are available from the literature [42,43], while only models for the effective piezoelectric coefficients of a mixture of piezoelectric inclusions in a non-piezoelectric matrix are documented [44]. However, one can easily realize, by the simple argument above, that the total piezoelectric displacement Δ*z*_cp_ would result as from Equation (2):

(2) $$\Delta z_{cp}=d_{c}E_{c}h_{c}+d_{p}E_{p}h_{p}=\frac{\left(d_{c}\varepsilon_{p}h_{c}+d_{p}\varepsilon_{c}h_{p}\right)}{\left(\varepsilon_{p}h_{c}+\varepsilon_{c}h_{p}\right)}V$$

and amounts to Δ*z*_cp_ ~ 1.1 Δ*z*_p_ for *h*_c_ = *h*_p_, with *d*_c_ ~ 100 pm/V (order of magnitude) for BaTiO_3_ and *d*_p_ ~ 20 pm/V for PVDF. For a thin polymer layer, e.g., *h*_p_ = 0.1 *h*, that for a 100nm diameter nanoparticle corresponds to 10nm, compatible with a typical gyration radius of a high molar mass polymer, one would obtain Δ*z*_cp_ that is only 1.75 times higher than Δ*z*_p_. This example shows how the maximum increase in the piezoelectric performance of the matrix material by addition of the ceramic inclusions is limited to a factor of about 2 in the case of the materials considered here. Incidentally, such a maximum is obtained when the polar directions of the two materials are parallel to each other, and point the same way; otherwise, different combinations should be considered, which could even decrease the total effect.

**Figure 5:**
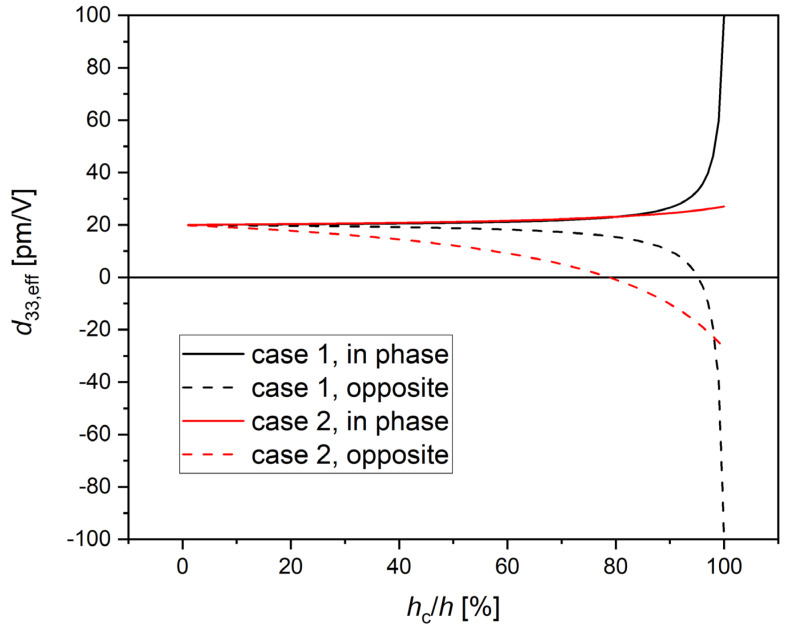
Dependence of the overall displacement for 1 V electric potential (*d*_33,eff_) from the relative thickness *h*_c_/*h* of the two layers (crystal and polymer) considered in the simple picture leading to Equation (2). Black curves (case 1): *ε*_c_ = 1000, *ε*_p_ = 10, *d*_c_ = 100 pm/V, *d*_p_ = 20 pm/V; red curves (case 2): *ε*_c_ = 50 [40], *ε*_p_ = 10, *d*_c_ = 27 pm/V [41], *d*_p_ = 20 pm/V. Solid curves: case of same orientation of the polar axes of crystal and polymer; dashed curves: case of opposite orientations.

The dependence of the overall piezoelectric performance as a function of the relative thickness of the two layers, with the values of dielectric and piezoelectric constants chosen for the above example (case 1), as well as with different values for the BaTiO_3_ nanoparticle consistent with characterizations from the literature (case 2), is shown in Figure 5. If the polar axes of the two materials are oriented likewise, increased piezoresponse is expected, while for opposite orientations, a decrease is found, which also has a zero and a phase inversion at some critical relative thickness (that is 95% for case 1, and 79% for case 2, as visible from Figure 5). This means that in principle, any value of *d*_33,eff_ is possible between 0 and some maximum piezoresponse, depending on the thickness ratio and the mutual orientation of the polar axes of the two materials.

The simple model in Figure 5 was considered here just to rationalize the general issue of the piezoelectric performance of composite materials. Nevertheless, planar geometry is not a valid approximation for the case of a scanning probe and of nanometer-size objects. In particular, in the simple planar geometry above, the mutual position of the two layers is not expected to influence the results, because of symmetry, whereas for a realistic tip/sample geometry, this is not the case. Indeed, the electric field obtained by using a sharp tip as one of the electrodes results as strongly inhomogeneous, being much higher close to the tip itself. On one hand, this provides the high imaging resolution peculiar to scanning probe microscopy, but on the other hand, the effect of the portion of material located closer to the tip may be enhanced compared to the rest of the material below. Therefore, scanning probe electric maps, including PFM, depend on the depth of the nano-inclusions within the matrix. This means that the measured effect of nanoparticles located near to the sample surface should appear more pronounced than the one caused by deeply buried particles, as far as scanning probes are concerned.

In the case of Figure 4, an isolated BaTiO_3_ nanoparticle was also present on the substrate, visible as the brightest spot next to the fiber on its left side in the piezoresponse map of Figure 4B (indicated by the arrows). From the corresponding line profile (Figure 4C), a high value of *d*_33,eff_ is recorded on the particle (around 60 pm/V, neglecting lateral overshoots due to slope effects, see Figure S4 and Table S4 of the Supplementary Materials), indicating that the surface of this particle is likely to be uncovered by the polymer, and therefore the internal electric field reduction above mentioned could be absent here (as from Equation (1), by using *h*_p_ = 0).

Because of the reduced symmetry of scanning probe systems, numerical simulations are usually needed to derive the dielectric properties of nanoparticles embedded in a polymeric matrix from measured image data [45]. Therefore, the location of nanoparticles in the polymeric matrix, as well as the orientation of the polar axes of both matrix and particles, are anticipated to concurrently influence the detected piezoelectric displacement, when investigated by PFM. This provides an interpretative key for the available PFM images.

As also evinced from the shown examples, the exploration of local piezoelectric properties provides a different kind of information with respect to macroscopic measurements of the same property. Whereas the global behavior of a nanofiber mesh could represent the final aim of research developments, especially for applications, knowledge of the sample structure and functionality at the nanometer scale helps to elucidate the reasons for some of the observed macroscopic behaviors, and to improve the production strategy to obtain the desired results. Additionally, local behavior could be more relevant than the global one, in those cases when the devised nanostructured system should be used as a support or host for different materials, as a gas or chemical sensor, or as a biological scaffold. For instance, piezoelectric fibers and/or nanoparticles could present a random orientation of their polar axes, so that the macroscopic efficiency of the system used as a pressure sensor or as an actuator could provide insufficient results, because of averaging effects. However, when a guest material is made to fill the spaces between the fibers, new interfaces are created, which could enable the desired functional behavior. For instance, if direct piezoelectric effect acts to stimulate cell growth or differentiation [5], only the local effect near to cell surfaces should be relevant, in contrast to the global piezoelectric effect resulting from an average over the whole scaffold. The latter consideration could be extremely important in biomedical applications. Therefore, availing oneself of methods to measure local piezoresponse, along with conventional ones to measure the global piezoelectric effect, would be crucial to develop and validate devices for those applications where the local cell–piezoelectric nanofiber contact is expected to promote specific tissue and organ signals, as seen in a piezoelectric cochlear implant, for example [6,9].

## 5. Conclusions

In conclusion, we have applied the CE-FM-PFM method to composite polymeric electrospun nanofibers, providing mapping of nanoscale piezoelectric behavior, with piezoelectric *d*_33,eff_ coefficient values consistent with the literature data for ferroelectric semicrystalline PVDF, and with the indication of fairly uniform poling along the fiber, although the presence of some ferroelectric domains was evidenced. Piezoelectric coefficients recorded near to embedded BaTiO_3_ nanoparticles can be diverse, by virtue of the quality of nano-inclusions, as well as of the orientation of their polar axes; in the case of our samples, the obtained local piezoelectric properties seemed limited. However, the main scope of the work was rather the confirmation that the CE-FM-PFM methodology could represent a convenient way to check the design and performance of piezoelectric nanostructured materials, for instance guiding towards the optimal electrospinning process parameters for producing piezoelectric devices and scaffolds.

## Data Availability

The data presented in this study are available in the present article and in the related Supplementary Materials.

## Supplementary Materials

The following supporting information can be downloaded at: https://www.mdpi.com/article/10.3390/polym14245379/s1, Figure S1: Thermogravimetric analysis (in air flux) of the composite nanofiber sample. Figure S2: Differential scanning calorimetry analysis of the composite nanofiber sample. Figure S3: PFM amplitude scan of an electrospun PVDF/BaTiO_3_ composite nanofiber transferred on a doped silicon substrate (Figure 2), with indications of three different regions: substrate (1), domain (2), nanofiber (3). Table S3: Average piezoresponse on three different regions indicated in Figure S3. Figure S4: Topography, resonance frequency shift (Δω) and piezoresponse (Δ*A*) scan of an electrospun PVDF/BaTiO_3_ composite nanofiber as deposited on the rotating electrospinning aluminum substrate, on which a BaTiO_3_ nanoparticle is also visible. Figure 4 was extracted from these topography and PFM amplitude full scans. Table S4: average piezoelectric coefficients on nanoparticle, nanofiber, and substrate in Figure S4.
