# Supplementary material for: Local Piezoelectric Response of Polymer/Ceramic Nanocomposite Fibers
Source: Polymers (Basel). 2022 Dec 8;14(24):5379. doi: 10.3390/polym14245379 (PMC9783531; doi:10.3390/polym14245379)
Supplement: Supplementary file 1 [file polymers-14-05379-s001.zip › polymers-2044665-supplementary.pdf]

Local Piezoelectric Response of Polymer/Ceramic Nanocomposite Fibers

Aurora Magnani [1], Simone Capaccioli [1,2,3], Bahareh Azimi [4,5], Serena Danti [4] and Massimiliano Labardi [2,*]

[1] Dipartimento di Fisica "Enrico Fermi", Università di Pisa, Largo Pontecorvo 3, 56127 Pisa, Italy
[2] CNR-IPCF, Pisa Unit, Largo Pontecorvo 3, 56127 Pisa, Italy
[3] CISUP, Centro per l'Integrazione della Strumentazione dell'Università di Pisa, 56126 Pisa, Italy
[4] Dipartimento di Ingegneria Civile ed Industriale (DICI), Università di Pisa, L. Lazzarino 1, 56122 Pisa, Italy
[5] Department of Translational Research and New Technologies in Medicine and Surgery, University of Pisa, 56126 Pisa, Italy
* Correspondence: labardi@df.unipi.it; Tel.: +39-050-2214322

Supplementary material

Figure S1. Thermogravimetric analysis (in air flux) of the composite nanofiber sample.

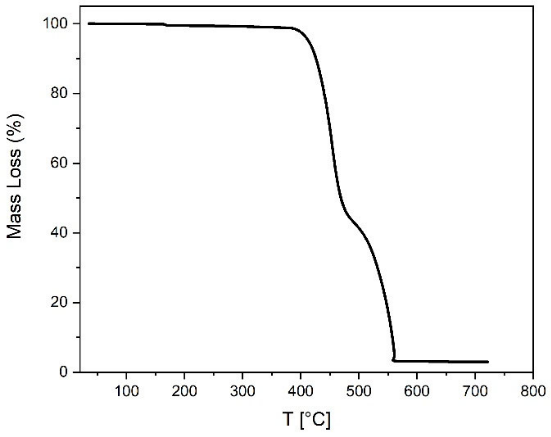

Figure S2. Differential scanning calorimetry analysis of the composite nanofiber sample.

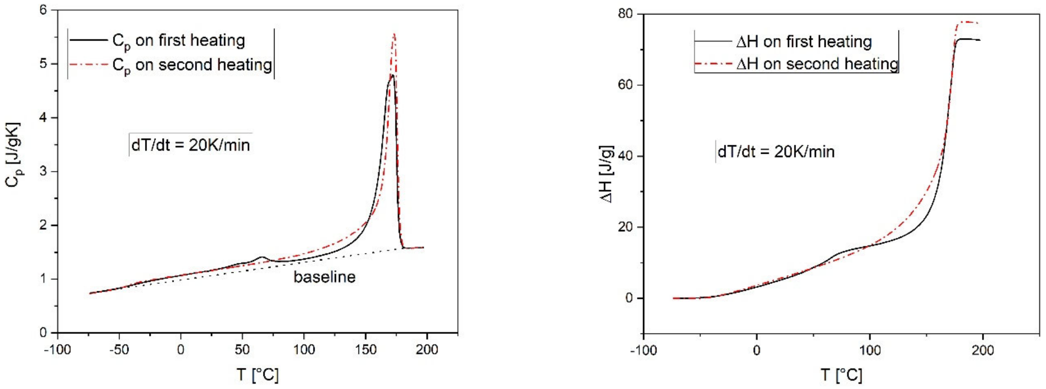

Figure S3. PFM amplitude scan of an electrospun PVDF/$BaTiO_3$ composite nanofiber transferred on a doped silicon substrate (Figure 2), with indications of three different regions: substrate (1), domain (2), nanofiber (3).

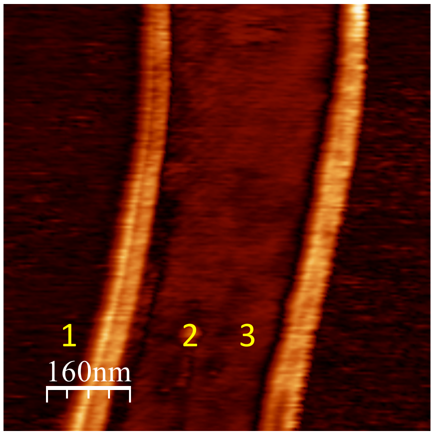

=======

Table S3. Average piezoresponse on three different regions indicated in Figure S3.

Position	Piezoelectric coefficient
Zone 1: substrate	6 pm/V
Zone 2: domain	31 pm/V
Zone 3: nanofiber	13 pm/V

Figure S4. Topography, resonance frequency shift (Dw) and piezoresponse (DA) scan of an electrospun PVDF/$BaTiO_3$ composite nanofiber as deposited on the rotating electrospinning aluminum substrate, on which a $BaTiO_3$ nanoparticle is also visible. Figure 4 was extracted from these topography and PFM amplitude full scans.

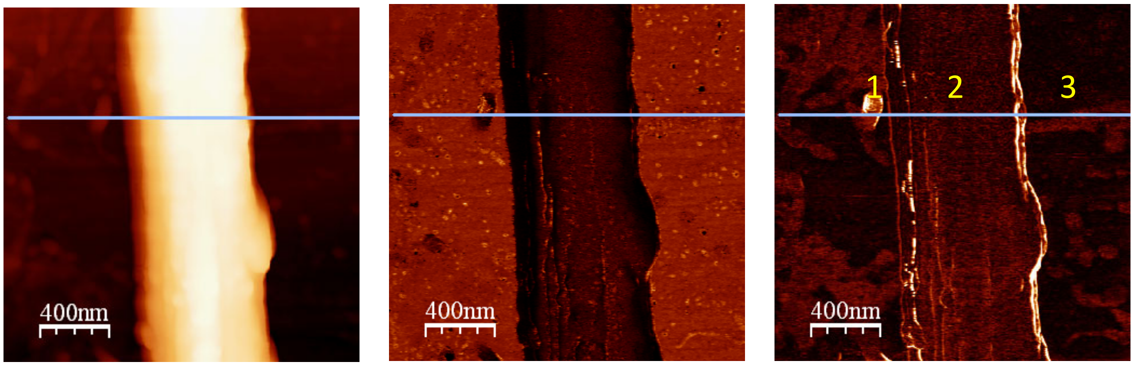

Topography Dw DA

Table S4. average piezoelectric coefficients on nanoparticle, nanofiber, and substrate in Figure S4.

Position	Piezoelectric coefficient
Zone 1: nanoparticle	60 ± 20 pm/V
Zone 2: nanofiber	17 ± 6 pm/V
Zone 3: substrate	7 ± 3 pm/V
